# Gastrointestinal and Autonomic Symptoms—How to Improve the Diagnostic Process in Panayiotopoulos Syndrome?

**DOI:** 10.3390/children9060814

**Published:** 2022-05-31

**Authors:** Aneta Zontek, Justyna Paprocka

**Affiliations:** 1 Students’ Scientific Society, Department of Pediatric Neurology, Faculty of Medical Sciences in Katowice, Medical University of Silesia, 40-752 Katowice, Poland; aneta114@gmail.com; 2 Department of Pediatric Neurology, Faculty of Medical Sciences in Katowice, Medical University of Silesia, 40-752 Katowice, Poland

**Keywords:** Panayiotopoulos syndrome, childhood occipital epilepsy, central autonomic network, autonomic seizure, fMRI, EEG

## Abstract

One of the most common epileptic disorders in the pediatric population is Panayiotopoulos syndrome. Clinical manifestations of this idiopathic illness include predominantly autonomic symptoms and dysfunction of the cardiorespiratory system. Another feature constitutes prolonged seizures that usually occur at sleep. It is crucial to differentiate the aforementioned disease from other forms of epilepsy, especially occipital and structural epilepsy and non-epileptic disorders. The diagnostic process is based on medical history, clinical examination, neuroimaging and electroencephalography—though results of the latter may be unspecific. Patients with Panayiotopoulos syndrome (PS) do not usually require treatment, as the course of the disease is, in most cases, mild, and the prognosis is good. The purpose of this review is to underline the role of central autonomic network dysfunction in the development of Panayiotopoulos syndrome, as well as the possibility of using functional imaging techniques, especially functional magnetic resonance imaging (fMRI), in the diagnostic process. These methods could be crucial for understanding the pathogenesis of PS. More data arerequired to create algorithms that will be able to predict the exposure to various complications of PS. It also concerns the importance of electroencephalography (EEG) as a tool to distinguish Panayiotopoulos syndrome from other childhood epileptic syndromes and non-epileptic disorders.

## 1. Introduction

About threepercent of the general pediatric population is affected by any type of epilepsy, which illustrates the high prevalence of this disorder in children [1]. Based on the epidemiology, etiopathogenesis and clinical manifestations, including the type of seizures and typical electroencephalographic features, a variety of epileptic syndromes may be recognized in the diagnostic process [1].

Self-limited childhood focal epilepsy is a group thatincludes, among others: Rolandic epilepsy (self-limited epilepsy with centrotemporal spikes), Panayiotopoulos syndrome (self-limited epilepsy with autonomic seizures), idiopathic childhood epilepsy of Gastatut (childhood occipital epilepsy), self-limited neonatal and infantile seizures caused by the development of the brain [1,2,3,4].

Panayiotopoulos syndrome, a common multifocal autonomic childhood epileptic syndrome, was primarily described by Chrysostomos Panayiotopoulos in 1988 and recognized by the International League against Epilepsy (ILAE) [2,5,6].

It was previously classified as an early-onset benign occipital epilepsy, but in the new ILAE Classification and Definition of Epilepsy Syndromes with Onset in Childhood was described as a self-limited epilepsy with autonomic seizures (SeLEAS) [2,6,7]. The prevalence of this benign epileptic syndrome is relatively high and is not significantly different between sexes [3,8].

The onset of PS ranges between 3–6 years, with the peak age of 4–5 years. It affects children with normal physical and cognitive development. Some reports state that even sixpercent of children between the agesof 1 to 15 might be affected by PS, and in the age group of 3 to 6 years old, even 13 percent [3,9].

According to a study by Weir, however, the incidence of PS may be lower than was previously indicated [10,11].

As of today, the etiopathogenesis of PS remains uncertain. Though there are indications of a genetic basis, the exact genes involved in the syndrome development have not been discovered [12,13].

The p.Phe218.Leu mutation of *SCN1A* (2q24.3; MIM #182389) gene is known as a genetic factor in general epilepsy with febrile seizures plus type 2 (GEFSP2; 2q24.3; MIM #604403). It also has been linked with PS and could be a determinant of the disorder’s severity [12]. *SCN1A* gene encodes voltage-gated sodium channels that are responsible for initiating and propagation of action potentials [13]. The majority of *SCN1A* gene mutations are de novo and seem to be associated with alternation of sodium channel function, mainly loss-of-function, although there are known mutations of this gene that are connected with gain-of-function. Pathogenic variants connected with Dravet syndrome are missense or truncating, in general causing total loss of function and severe phenotype. GEFS+ is associated with moderate alternation of sodium channel function that results in a milder phenotype [14]. It is worth mentioning that mutations of the *SCN1A* gene may lead to various functional changes in sodium channel activity and could lead to hyperexcitability as well as hypoexcitability of nerve cells. Gain-of-function mutations are connected with familial hemiplegic migraine or early infantile encephalopathy [15,16].

In some cases, it is difficult to identify the main effect on the protein activity because mutations can lead to increased, decreased, or even mixed bothloss- and gain-of-function variants. The heterogeneity results in serious challenges in the treatment processand potential implementation of sodium channel blockers [12,13,14,15,16,17,18,19]. According to studies, mutations of the *SCN1A* gene related to PS lead to moderate alternation of the voltage-gated sodium channel, probably resulting in a decrease inits function; however, various additional factors, including environmental or epigenetic components, could have an impact on phenotype development.

Predominantly and previously autonomic manifestations are connected with ictal epileptic discharges that later transmit to higher-level regions of the central nervous system (CNS) [20].

PS can mimic various epileptic and non-epileptic disorders. Specific challenges can occur during the diagnostic process that can result in misdiagnosis and implementation of inappropriate treatment methods [20,21]. Moreover, patients who are affected by self-limited epilepsy with autonomic seizures are at risk of developing specific neurocognitive complications [22,23,24,25]. By using EEG, it is possible to distinguish PS from other epileptic syndromes and non-epileptic diseases, although implementing functional neuroimaging techniques could improve the accuracy of the diagnostic process and allow the identification of specific complications [26,27,28,29].

The main purpose of this review is to discuss the potentiality of implementing noninvasive and repeatable neuroimaging techniques, especially functioning magnetic resonance imaging (fMRI), in the diagnostic process to predict neurocognitive consequences or increased susceptibility to some internal complications, including sudden unexpected death in epileptic patients (SUDEP). What is more, in this article, we would like to underline the diagnostic challenges in patients with PS due to the broad spectrum of clinical manifestations and concerntherole of EEG as an investigation toolin the diagnostic process. According to current issues, it is crucial to bear in mind usingvarious diagnostic techniques to improve the accuracy of the final diagnosis as well as to implement appropriate management and avoid unnecessary treatment methods.

## 2. Results

### 2.1. Clinical Manifestations

#### 2.1.1. Autonomic Symptoms

The most characteristic clinical features of PS are associated with the autonomic system include nausea, retching and vomiting, pupils’ dilation-mydriasis or constriction-miosis, excessive salivation, turning pale, cyanosis or flashing, deviation of head and eyes, urine or/and feces incontinence, sweating, cardiorespiratory system dysfunction, heart rhythm abnormalities such astachycardia, altered thermoregulatory responses or abnormal intestinal contractions. The characteristic emetic triad (nausea, retching, vomiting) is described as ictus emeticus. It is essential as well that vomiting appears in almost 75 percent of PS episodes (Table 1) [5,30,31,32].

Ictus syncope is another common clinical manifestation that occurs in Panayiotopoulos epilepsy when patients present with unresponsiveness and flaccidness [1,3,30].

Seizures in PS are usually observed during night sleep or daytime naps. Around 50% of seizures are partial or generalized tonic. It is crucial that approximately fifty percent of seizures are prolonged, last more than 30 min and often evolve into autonomic status epilepticus [3,9]. The average episode duration in this type of PS is 2 h, whereas other PS patients present with a shorter duration of seizures -ca. 9 min [3].

The typical seizure starts with nausea and vomiting, which is later accompanied bydifferent grades of loss of consciousness [3].

#### 2.1.2. Other Clinical Manifestations

Other clinical signs and symptoms of SeLEAS include headaches, auras equivalent and ictal eye or head deviation. PS can also manifest as speech disability, unilateral contractions of facial muscles, oropharyngolaryngeal movements or myoclonia (Table 1) [1,3,30].

### 2.2. Neurocognitive Dysfunctions and Possible Internal Complications, including SUDEP in Patients withPanayiotopoulos Syndrome

According to the research by Fonseca Wald, patients with SeLEAS are prone to manifest dysfunction, especially in verbal and visual memory.Children with PS gain standard notes in IQ scores;however, they usually seem to be on the lower end of average mental ability [22].

A study of Akca Kalem’s results hasdemonstrated that patients with PS are more prone to cognitive disabilities than children with Gastaut syndrome. In comparison to GS, people affected by SeLEAS have problems with reading and their intelligence quotient scores are remarkably lower than the results of children with GS [23].

According to current research, there is no significant difference in the prevalence of behavioral dysfunctions between both research groups and healthy control subjects [23].

Prolonged seizures and status epilepticus could have an impact on the development of cognitive disabilities in children with PS by excitotoxicity, inflammatory responses and ischemic lesions resulting in the disarrangement of neuronal circuits [33].

Visual–perceptual dysfunctions in children with PS may be associated with spike discharges mainly localized in the occipital area; however, the wide spectrum of dysfunctions observed in this epileptic syndrome could be associated with multifocal epileptiform activity and be a consequence of involving various brain regions in epileptiform discharge propagation [34].

The main purpose of performing the diagnostic process using conventional neuroimaging methods such as CT or MRI seems to be, in general, excluding potential brain lesions such as CNS tumors, cerebral infarction or intracranial hematoma that could be responsible for observed clinical symptoms [28].

The idiopathic character of presented syndrome means that structural neuroimaging results are normal without any suspected abnormalities except for some coincidental brain lesions such asarachnoid cysts or focal encephalomalacia focal detected by imaging investigation according to this study by Yalçin [35].

Functional neuroimaging diagnostic methods such aspositron emission tomography (PET) and functional MRI could investigate the possible pathomechanism of various neurological disorders, including childhood idiopathic epileptic syndromes [34]. According to the studies, the pathogenesis of focal epilepsy is associated with a brain network’s abnormality rather than a single CNS area’s dysfunction [36,37]. Identification of an epileptogenic network described as a structure located in the CNS that abnormal neuronal activity leads to epilepsy propagation provides an understanding of the specific syndrome’s mechanism SeLEAS in the pediatric population does not require using invasive procedures during the diagnostic process because of its benign character; however, there is strong evidence that children who are affected by PS are at risk of developing neurocognitive dysfunction in the future [22,23,24,25,38].

Taking into consideration the presented results, it seems to be essential to detect this specific group of children with PS who are more susceptible to suffering from various neurological disabilities. Another crucial purpose of investigating functional methods of neuroimaging is to identify specific groups of patients who are at risk of SUDEP. The pathogenesis of this serious complication has still not been clearly defined, although there is evidence of autonomic circuit dysfunction as a potential mechanism in SUDEP development [20,39].

The role of functional neuroimaging in SUDEP’s prediction—one of the most serious internal complications of SeLEAS is sudden unexpected death in epileptic patients. The frequency of this severe outcome is not high, although it is crucial to bear in mind this possibility in the patient’s history [39,40,41].

Studies have shown that patients who are at higher risk of SUDEP and who died due to presented complications were characterized by altered, less organized subunits of central autonomic circuits responsible for controlling the cardiorespiratory responses [39,40]. According to the Verrotti study, the occurrence of SUDEP in children with PS is about 1 out of 200 cases with SeLEAS, so the prevalence of present complications in this group is 0.5 percent. [9].

### 2.3. Functional Neuroimaging

Results of other studies suggest that pathogenesis and clinical manifestations of self-limited epilepsy with autonomic seizure are associated with brain structure maturation and hyperexcitability of specific autonomic circuits belonging to the CAN. Moreover, the characteristic sequence of clinical symptoms with predominantly autonomic manifestations seems to be associated with decreased seizure threshold characteristics for autonomic neurons in comparison to specific cortical regions connected with particular motor or sensory responses. Primary epileptogenic activity may spread to higher-order brain areas and result in focal seizures that could evolve into generalized seizures. The role of the central autonomic network and possible dysfunction of this area could be recognized by using methods of functional neuroimaging, including positron emission computed tomography (PET) and functional magnetic resonance imaging (fMRI) [34,42,43,44,45].

Functional methods of neuroimaging include PET and fMRI. Both procedures are noninvasive and repeatable; however, the main advantage of using an fMRI that is based on the detection of changes in blood flow and deoxyhemoglobin concentration (BOLD—Blood Oxygen Level Dependent) over PET in the pediatric population is the lack of exposure to radioactive substances [46].

Presented procedures are usually used in the presurgical evaluation, although identification of blood flow changes in specific brain regions can suggest cognitive or motor impairment. In comparison to PET, fMRI detects cerebral perfusion changes indirectly by distinguishing oxyhemoglobin and deoxyhemoglobin concentration [46].

Brain electrical activity is connected with the higher extraction of blood oxygen. A comparison of magnetic resonance signal change is required. The essential issue in differentiating between average brain activity and reorganization in neuronal networks is creating specific maps with high localization accuracy [26,47,48].

Using resting-state fMRI or task-related fMRI as well allows the detection of functional connectivity and seems to be a very useful procedure to identify the pathogenesis of specific epileptic syndromes by detection of significant changes in specific neuronal circuits activity. In order to internalize psychological or behavioral comorbidities in SeLEAS that could have a significant impact on the general activities, these techniques should be implemented as a potential tool in the diagnostic process [26,27,48,49].

Moreover, the integration of fMRI and EEG study allows fordetection and distinguishing whether revealed abnormalities in neuronal networks are connected with interictal activity [49].

The fMRI technique seems to be a valuable and useful tool to improve accuracy and precision during the diagnostic process as well as to detect comorbidities and further cognitive and internal complications;there are some limitations to using this procedure, including availability, price and a lack of data [46,50].

### 2.4. Central Autonomic Network

**Figure 1:**
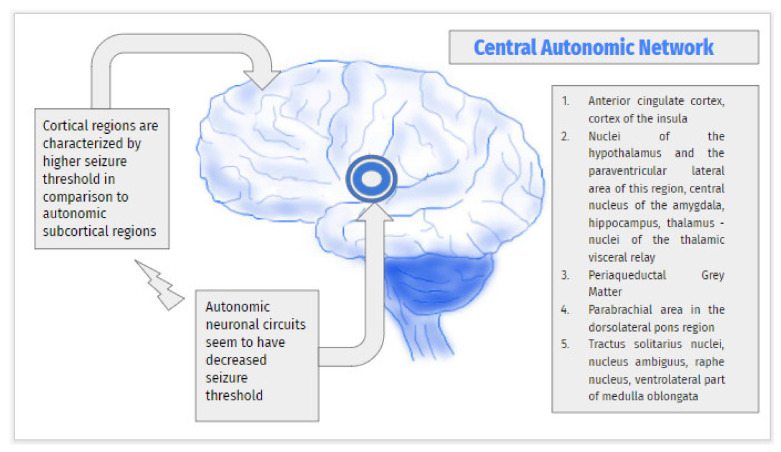
This figure shows the main components of the central autonomic network according to Benarroch and demonstrates that cortical regions are characterized by higher seizure thresholds in comparison to autonomic subcortical regions. A lower seizure threshold of subcortical components results in predominantly autonomic symptoms with further focal cortical seizures due to propagation of epileptiform discharges.

The central autonomic network (CAN) is a structure divided into three principal components in relation to the region of the CNS. It is composed of extra-hypothalamic nuclei and integral parts of the hypothalamus. It is responsible for controlling and regulating the influence of the autonomic nervous system on the neuroendocrine, cardiovascular, respiratory and gastrointestinal systems as well as on the thermoregulatory mechanisms. Moreover, this formation is also associated with individual responses to pain, behavioral and neurocognitive functions. Specific components CAN manifest their impact on the regulation of sympathetic activity, while other parts play a key role in controlling mechanisms connected with the parasympathetic nervous system [20,34,40,47,51,52,53] (Figure 1).

Structures responsible for sympathetic responses includethe locus coeruleus, whichcontains noradrenergic neurons and ventrolateral medulla oblongata. Components that have an influence on parasympathetic mechanisms include the parabrachial region, nucleus ambiguus, nucleus of the vagus, corpus amygdaloideum and periaqueductal gray matter. Other parts have an impact on both principal elements of the autonomic nervous system. Presented regions are involved in creating specific autonomic circuits characterized by interconnection and internal integrality as well. Each neuronal network is involved in regulating basic functions and maintaining homeostasis [45,51,54].

Areas associated with vomiting include the area postrema, which is described as the chemoreceptor trigger zone. The dorsal vagal nucleus, the nucleus of the solitary tract, parvocellular reticular formation and ventral respiratory groups are responsible for initiating and controlling the motor components of vomiting [51].

Autonomic symptoms are related to hyperactivation of CAN structures, as indicated in the study by Saito, in which the epileptogenic zones of PS were shown to be located in the area of the calcarine sulcus and parieto-occipital sulcus [55]. The predominance of the occipital region in most cases, although an abnormal neuronal activity was also present in other CNS areas.Clinical symptoms and discharge location did not correlate, contrary to the presence of correlation between the patient’s age and location of epileptogenic zones. The epileptiform activity manifested as frontal spike discharges was significantly higher in a group of older patients than in younger children with spikes detected in the area of parieto-occipital, calcarine and central sulcus [38,55].

Results of other studies suggest that pathogenesis and clinical manifestations of self-limited epilepsy with autonomic seizure are associated with brain structure maturation and hyperexcitability of specific autonomic circuits belonging to the CAN [4]. Moreover, the characteristic sequence of clinical symptoms with predominantly autonomic manifestations seems to be associated with decreased seizure threshold characteristics for autonomic neurons in comparison to specific cortical regions connected with particular motor or sensory responses. Primary epileptogenic activity may spread to higher-order brain areas and result in focal seizures that could evolve into generalized seizures (Figure 1). The role of the CAN and possible dysfunction of this area could be recognized by using methods of functional neuroimaging [42,43,44,45].

### 2.5. Differential Diagnosis, the Diagnostic Process and Treatment Methods

Differential diagnosis includes other forms of epileptic syndromes such asRolandic epilepsy, described as self-limited epilepsy with centrotemporal spikes, and childhood occipital visual epilepsy named Gastaut syndrome [56].

In casesthat are definitely non-specific to epileptic disorders, clinical symptoms of autonomic status epilepticus in PS could be misclassified and incorrectly diagnosed as a non-epileptic condition. The epileptic explanation is suspected by electroencephalography features and motor ictal symptoms presented after seizure episodes [21,56]. It is essential to distinguish PS from non-epileptic disorders due to the similarity of clinical features. These conditions include headache syndromes such asmigraine or parasomnias such assomnambulism [21,56]. Primary signs and symptoms such as vomiting or nausea could suggest gastrointestinal illnesses such as GERD (gastroesophageal reflux disease), gastrointestinal inflammatory diseases, CVS (cyclic vomiting syndrome) or increased intracranial pressure due to brain tumors [21,56,57].

Misdiagnosis includes syncope on both vasovagal and neurocardiogenic basis. Differential diagnosis embraces cardiomyopathies, arrhythmias or long QT syndrome [21,58]. Moreover, in the general infant population, PS may mimic various metabolic disorders [21].

The diagnostic process is mainly based on medical history and presented symptomsand should be confirmed by EEG findings. Performing an EEG seems to be the gold standard in the diagnostic process and helps to exclude non-epileptic diseases, as well as it could be a tool to distinguish PS from other childhood epileptic syndromes. On the other hand, physicians should be aware in cases of insufficient specificity of the pattern in the observed changes [58].

EEG results reveal high amplitude focal spikes, intensified by sleeping and increasing a respiration rate, mainly in the area of occipital lobes. Other kinds of EEG findings consist of multifocal spikes. About 70 percent of affected children have EEG abnormalities associated with occipital spikes or occipital paroxysms. Posteriorly located spikes are mostly bilateral and characterized by synchronicity. The wave complexes are characterized by high amplitude [1,2,5,59,60].

Interictal spikes abnormalities are observed in more than 70 percent of cases. Performing EEG in the intervals between clinical attacks shows multifocal spikes with occipital predominance. The background activity demonstrates no abnormalities. EEG during sleep reveals discharges in the extra-occipital areas (mainly frontal parts, centro- and frontotemporal regions) in 40 percent of patients [59,60].

According to the research, sequences of EEG features do not correlate with specific signs and symptoms which are characteristic ofSeLEAS [59].

EEG could be the tool to determine the form of epilepsy by showing characteristic patterns of discharges and distinguishing PS from other types of childhood epileptic syndromes and non-epileptic disorders such asparasomnias or acute encephalopathy (Table 2 and Table 3) [11,21,29,61,62,63,64,65,66,67,68,69,70,71,72,73,74,75,76].

Because of the benign nature of PS, anti-epileptic treatment is not always required [8,77]. In the case of prolonged seizures that last more than 10 min or status epilepticus some pharmacological treatment methods should be implemented [8]. Adults are not affected by this syndrome. Prognosis in this syndrome is very good, although 21 percent of cases are prone to evolve to other kinds of childhood or juvenile epileptic syndrome, including juvenile myoclonic epilepsy or Rolandic epileptic syndrome [10,78,79]. Consequences of misdiagnosis include inadequate treatment methods and unnecessary stress on patients and their parents. It is crucial to bear in mind these possibilities during the diagnostic process in order to avoid unnecessary procedures that can result in unwanted side effects [20].

### 2.6. The Discussion on Diagnostic Challenges in Patients with PS

Taking into consideration the broad spectrum of clinical manifestations in patients with PS, there is no doubt that the complex diagnostic process is connected with using a variety of both invasive and non-invasive procedures. The diagnostic challenges could be resolved by applying selected procedures characterized by repeatability and non-invasiveness. It is essential to avoid implementing unnecessary management and overtreatment, but in some cases, there are difficulties in recognizing the definitive diagnosis. The first point of the whole investigation process should be based on the patient’s medical history, including familial health history and performing the physical examination. All eventual abnormalities and dysmorphic features should be evaluated. The second part concerns the additional tests, including laboratory analysis, imaging techniques—CT, MRI, ultrasonography, endoscopic procedures—eventually esophagogastroduodenoscopy or capsule endoscopy, diagnostic for cardiovascular conditions—electrocardiography—ECG Holter monitoring, echocardiography, functional cardiac testing. What is more, precise neurological evaluation is required due to the quite high incidence of epileptic syndromes in the general pediatric population (Table 4.) [20,21,80,81]. Signs and symptoms should be evaluated as a coexistence of clinical manifestations, not only as distinct abnormalities.

According to the study by Chauvel, the evaluation of the semiology of epileptic seizures plays a key role in the preliminary diagnostic management. Detection of specific neuronal circuits, configuration and dynamic interconnection of their components may improve the whole diagnostic process in patients with epilepsy and achieve accuracy in the evaluation of potential complications or comorbidities. The stereoencephalography (SEEG) is an invasive monitoring technique that can reveal correlations between anatomical components associated with epileptic discharge occurrence and propagation and their electro-clinical manifestations [81]. Generally, this procedure is used to identify the epileptogenic zones in the presurgical intracranial investigation in epilepsy surgery [82]. Due to self-limitation and generally good outcomes in patients with PS, techniques such as SEEG usually are not implemented. The further step to upgradingthe diagnosis of the aforementioned epileptic syndrome is to implement accurate procedures in the correct sequence.

The case study of Gaur reveals the importance of careful investigation of a patient’s medical history; however, this case report concerns frontal-lobe epilepsy in comparison to non-epileptic seizures;it could be a valuable example of accuracy in neurological diagnostic management even if patients present unspecific features, suggesting psychiatric or internal causes [83].

In the diagnostic process, psychological non-epileptic seizures, also described as pseudoseizures, should be taken into consideration as an explanation reached by the elimination process. PNES are characterized by transient events thatclinically resemble epileptic seizures but without the occurrence of EEG abnormalities. According to studies, these kinds of seizures could be associated with alterations in the controlling and consolidatingof neuronal assemblies [84,85].

To sum up, although it is crucial to determine potential severe disorders and harmful dysfunctions, the balance between necessary management and invasiveness is required.

## 3. Conclusions

Performing EEG can distinguish PS from other epileptic and non-epileptic disorders; however, it does not seem to be enough to detect specific comorbidities and cognitive complications that may occur in the future. Results of EEG sometimes may be indecisive, especially during interictal activity. SeLEAS is characterized by multi symptomaticity and it is prone to misdiagnosis. A broad spectrum of symptoms and nonspecificity of clinical manifestation may lead to the implementation of various unnecessary procedures and delay an appropriate final diagnosis.

Moreover, due to high frequency among the pediatric population and potential cognitive impairments, it seems to be useful to implement special noninvasive and repeatable functional neuroimaging techniques to improve the whole diagnostic process. Patients with PS are at risk of developing various cognitive dysfunctions, especially visual memory disabilities. They also gain generally lower results in full-scale IQ. It seems to be crucial to identify these children who are prone to developing these abnormalities, in the case of the value of prediction, educational and rehabilitation procedures as well. Another potential benefit is connected with reduced prolonged diagnostic delay; however, to create a specific algorithm based on data collected during functional neuroimaging procedures implementation, further research is required.

## Figures and Tables

**Table 1:** The table demonstrates the main autonomic and other clinical manifestations characteristic ofPS.

Autonomic Symptoms	Other Clinical Manifestations
nausea, retching and vomiting—ictus emeticus triad	loss of consciousness
turning pale, cyanosis or flashing	ictal eyes or head deviation
pupils dilation (mydriasis) or constriction (myosis)	loss of consciousness
	tonic seizures
	partial or generalized eneralized clonic movements
excessive salivation	headaches
urine or/and feces incontinence	disability of speech
heart rhythm abnormalities (tachycardia/bradycardia)	myoclonia
respiratory system abnormalities	oropharyngeal movements
altered thermoregulatory responses sweating	unilateral contraction of facial muscles
abnormal intestinal contraction	auras equivalent

**Table 2:** This table compares EEG patterns characteristic ofselected childhood epileptic syndromes.

Childhood Epileptic Syndrome	EEG Characteristic Patterns	EEG Changes Cont.	Additional Features and Information
Rolandic epilepsy self-limited epilepsy with centrotemporal spikes	the epileptiform activity associated with the Rolandic area cortex	biphasic unilateral spikes	most common epileptic disorder in pediatric population
	slow waves spikes from the contralateral to the symptoms hemisphere ictal activity	centrotemporal high-amplitude wave discharges (100 to 300 µV)	
		normal background activity—interictal activity	
Gastaut-Lennox epilepsy late-onset occipital lobe epilepsy		frontal high- amplitude spikes	severe character of epilepsy
	slow background and diffuse slow spike slow wave activity (<2.5 Hz)	focal or generalized discharges paroxysms and abnormal waveforms—interictal activity	nocturnal EEG, especially during the non-REM period useful in identifying characteristic EEG patterns
Idiopathic photosensitive occipital lobe epilepsy	ictal discharges located in the occipital regions	background activity generally normal	discharges and seizures are triggered by light
		Rolandic spikes observed in some cases	common visual symptoms
Juvenile absence epilepsy	diffuse 3–6 Hz generalized spike	wave pattern +/−polyspikes	

**Table 3:** This table compares characteristic EEG related to selected non-epileptic disorders. Characteristic for PS.

Non-Epileptic Disorders	EEG Characteristic Patterns	Additional Features and Informations	Additional Features and Information
Migraine	migraine with aura—delta waves activity (slower rhythm patterns and epileptic discharges) alpha rhythm asymmetry characterized by inter hemisphericity	coexistence with epileptic syndromes	EEG abnormalities presented mainly during attacks of migraines with auras
	migraine without aura—in general normal activity, in some cases slowing of the activity in the posterior brain regions	EEG in general demonstrates normal activity	
Encephalitis	diffuse EEG changes and slowing of the background activity	detection of various forms of epileptic activity in encephalitis is possible by using continuous EEG	
Encephalopathy	hypoxic ischemic encephalopathy -continuous and reactive EEG pattern -continuous and unreactive EEG pattern -burst suppression in the background EEG pattern epileptogenic encephalopathy—burst suppression/hypsarrhythmia (early childhood) multifocal/generalized discharges slowing of the background (later childhood/adolescence)		In comparison to PS, convulsive seizures are greater than 15 min
	Reye syndrome—diffuse slowing of the background, sharp/spikes wave activity Hepatic encephalopathy—in the pediatric population—epileptiform discharges/diffuse slowing triphasic waves activity—acute liver failure		Seizures in patients with PS able to be averted by smaller doses of midazolam in comparison to encephalopathic ones
	Specific encephalopathic syndromes: -Tay-Sachs disease—EEG pattern reveals fast central spikes -Maple syrup urine disease—comb-like rhythm—7–9 Hz central activity -Sialidosis type 1—vertex sharp waves -Infantile neuroaxonal dystrophy—16–24 Hz amplitude discharges		EEG can provide informations about severity of encephalopathy
Non-REM related sleep disorders	hypersynchronous delta waveforms (characteristic for somnambulism)	higher arousal index	EEG changes are more frequent in the pediatric population in comparison to the adults
	slow wave sleep activity		

**Table 4:** This table demonstrates clinical manifestations in PS, potential disorders that present similar clinical features and may mimic SeLEAS. Proposed diagnostic procedures are introduced.

Clinical Manifestations in PS	Potential Differential Diagnosis	Proposed Diagnostic Procedures
nausea, vomiting and retching		laboratory tests (electrolytes, glycemia, morphology, toxicology tests etc.)
	gastrointestinal disorders (gastroenteritis, gastroesophageal reflux disorder, acute abdominal syndrome) infectious diseases metabolic disorders toxicological causes cyclic vomiting syndrome brain tumor (increased intracranial pressure) psychogenic vomiting with nausea psychiatric disorders (bulimia) pregnancy	endoscopic procedures abdominal ultrasound
		abdominal CT neuroimaging
loss of consciousness	syncope: -vasovagal, -neurally mediated syncope, -cardiovascular factors (cardiomyopathies, pulmonary hypertension, right or left ventricular outflow tract obstruction tachyarrhythmias)	electrocardiography, Holter ECG monitoring, echocardiography cardiac MRI
		EEG
heart rhythm alterations	brady/tachyarrhythmias	Holter ECG monitoring
headache	migraine	
	tension headache	physical examination medical history
	brain tumor	
turning pale cyanosis flashing	hypotonia obstruction of the airway pulmonary hypertension superior vena cava syndrome neuroblastoma carcinoid	physical examination USG thoracic and abdominal CT PET laboratory tests (urinary catecholamine metabolites 5-HIAA level)
mydriasis/miosis	drugs/medications intake neurological dysfunction (increased ICP, intracerebral tumor, subarachnoid hemorrhage)	toxicology tests neuroimaging
nocturnal seizures/seizures during sleep	sleep disorders (especially non-REM sleep disorders)	polysomnography
		video-EEG
focal cortical seizures	focal neurological deficits due to: -intracerebral tumor -ischemic stroke -stroke-episodes other childhood epileptic syndromes	CT imaging MRI imaging EEG video-EEG
generalized seizures	encephalopathies encephalitis meningitis other childhood epileptic syndromes neurometabolic disorders fever seizures PNES	EEG, neuroimaging, laboratory tests (sodium, potassium, glycemia, ammonia, ketone bodies, urea, creatinine, C-reactive protein, cerebrospinal fluid analysis etc.)
speech disability	afasia dysarthria due to stroke or stroke-like episodes	neuroimaging
head/eyes deviation	brain tumor other types of epileptic syndrome	neuroimaging EEG
